# Application of Silica-Aerogel-Fibre-Based Thermal Renders for Retrofits in Building Walls: A Comparative Assessment with Benchmark Solutions

**DOI:** 10.3390/gels9110861

**Published:** 2023-10-30

**Authors:** Marco Pedroso, José Dinis Silvestre, M. Glória Gomes, Jéssica D. Bersch, Inês Flores-Colen

**Affiliations:** 1 Civil Engineering Research and Innovation for Sustainability (CERIS), Departamento de Engenharia Civil, Arquitetura e Ambiente (DECivil), Instituto Superior Técnico (IST), Universidade de Lisboa, Av. Rovisco Pais, 1049-001 Lisbon, Portugal; marco.pedroso@tecnico.ulisboa.pt (M.P.); jose.silvestre@tecnico.ulisboa.pt (J.D.S.); maria.gloria.gomes@tecnico.ulisboa.pt (M.G.G.); jessica.d.bersch@tecnico.ulisboa.pt (J.D.B.); 2 Núcleo Orientado para a Inovação da Edificação (NORIE), Programa de Pós-Graduação em Engenharia Civil: Construção e Infraestrutura (PPGCI), Universidade Federal do Rio Grande do Sul (UFRGS), Av. Osvaldo Aranha, 99, 7th Floor, Porto Alegre 90035-190, Brazil

**Keywords:** silica aerogel, fibre, thermal insulation, thermal render, retrofit, facade, optimum insulation thickness, life cycle savings, payback periods, environmental impacts

## Abstract

The current climate change context raises the demand for reducing energy and environmental impacts while keeping an economic balance and building users’ comfort. Thermal insulation solutions are potential allies in ensuring the adequacy of existing buildings for challenging sustainability requirements. In this scenario, silica-aerogel-fibre-based thermal renders are innovative solutions for which integrated approaches still lack information, and they should be compared with benchmark multilayer solutions, such as those based on expanded polystyrene (EPS), extruded polystyrene (XPS), mineral wool (MW), and insulated corkboard (ICB), to evidence their prospective economic, environmental, and energy benefits. This paper quantifies the optimum insulation thicknesses, life cycle savings, payback periods, and environmental impacts of innovative thermal renders compared to conventional thermal insulation materials when applied as a retrofit in existing facade walls. The results show that cost-optimised thermal renders with sisal fibres led to the best overall performance. Higher heating needs led to higher optimum render thicknesses and life cycle savings. With a 0.02 m thickness, aerogel-fibre-based thermal renders outperformed other materials in terms of heating-degree days (HDD) from 1000 °C·day onwards; they can save approximately EUR 60∙m^−2^, 1000 MJ∙m^−2^, and 100 kg CO_2_ eq∙m^−2^ while presenting a U-value 13% lower throughout their 30-year lifetime when compared with the second-best multilayer solution with XPS.

## 1. Introduction

In the context of the current climate change concerns, the need for achieving energy-efficient buildings and thus promoting retrofit actions seeking to ensure resilience in the built environment, with lower resulting impacts, has become crucial [1]. Facades act as decisive systems regarding energy demands and outdoor and indoor thermal comfort [2] since they significantly affect heating and cooling needs [3]. Concerning existing buildings and their facades, there are challenges related to retrofitting measures to promote sustainability and mitigate climate change [4]; therefore, research is needed on this topic, especially taking into account the massive available building stock [5].

Among the strategies for retrofitting a building envelope, the inclusion of shielding systems, the replacement of transparent components, and, lastly, the insulation of the opaque components may be highlighted [6]. Thermal insulation contributes considerably to decreasing heating and cooling energy consumption [7]. Expanded polystyrene (EPS), extruded polystyrene (XPS), and mineral wool (MW) [8,9,10,11] are materials that are commonly studied to improve insulation in building walls. Moreover, insulation corkboard (ICB) is referred to as an insulation material [12]. Furthermore, thermal insulation plasters are being addressed as interesting alternatives for use in facades when seeking lower costs than conventional materials in conjunction with potential high thermal efficiency [13].

Regarding thermal plasters, aerogel-based renderings are promising energy-efficient composites for retrofits in uninsulated building envelopes [14,15]. Silica aerogel is highly porous and has low thermal conductivity [16] (0.015 W/(m·K), as reported in [17]) and low density [18]. Several studies are being developed considering the incorporation of nanometric silica aerogel as an aggregate within thermal insulation renders. Physical, mechanical, and microstructural properties; porosity; and hygrothermal behaviour, among others, have already been addressed [18,19,20,21].

A suitable compromise must be sought among the effects of aerogels when added into renders, enabling, for instance, good thermal performance associated with adequate mechanical properties [22]. In this context, fibres have been added to aerogel-based thermal renders for which satisfactory formulations are sought [20]. Mazrouei-Sebdani et al. [23] explained that silica aerogels have weak inter-particle connections, which characterise them as brittle, and, therefore, the combination of these innovative materials with fibres may actually enhance mechanical and insulation performance in addition to supporting their practical application.

Economic and energy life cycle assessments of aerogel-based thermal renders were carried out by Garrido et al. [24], leading to the conclusion that in the case of retrofits, significant energy and economic savings may be achieved during the remaining service life of buildings due to the application of thermal renders, despite their high initial cost. In addition, the incorporation of aerogel in already-existing building materials, such as mortars, can lower its production cost and may also contribute to its acceptance in the construction sector [22].

Although conventional insulation materials have already been compared considering their performance [11], to the authors’ knowledge, there is still a scientific gap concerning the comparison of usual benchmark insulation solutions with innovative aerogel thermal renders. Such a comprehensive assessment lacks information, especially regarding an integrated approach including environmental, economic, and energy-related points of view.

The importance of an integrated approach may be corroborated by the study of Lamy-Mendes et al. [22], whose conclusions pointed to a significant reduction of up to 65% in the emission of greenhouse gases in the use of aerogels when compared to XPS, EPS, and foamed polyurethane (PU). Huang et al. [25] verified that aerogel blankets provided the minimum optimum insulation thickness for use in a typical Chinese building compared to XPS, EPS, PU, and glass fibre (GF); furthermore, aerogel offered the fastest reduction in greenhouse gas emissions with increasing thicknesses [25]. Moreover, regarding retrofitting, the use of aerogel-containing thermal renders instead of recurrently used thermal insulator materials (like EPS and XPS) practically benefits from its application method over the substrate surface, which may be rough and uneven, and, even so, can be applied with a continuous thermal insulation layer with gaps and joints reasonably filled [26].

Therefore, in this context, the present paper aims to contribute to the in-depth understanding of aerogel-fibre-based thermal renders to be used in the retrofitting of existing buildings compared to benchmark solutions, seeking better environmental, economic, and energy behaviour within the context of climate change. Optimum insulation thicknesses, life cycle savings, payback periods, and the environmental impacts of the innovative thermal renders were quantified and assessed against the conventional thermal insulators EPS, XPS, ICB, and MW and also against a commercial thermal render containing cork granules (TR cork) in multilayer facade systems. 

Previously studied aerogel-containing renders with aramid and sisal fibres were considered for an integrated investigation. This research focused on numerical simulations regarding the application of the thermal renders as retrofits in facade building walls located in the Azores and Bragança regions in Portugal, which have the most distinct climate characteristics within the whole country. Furthermore, the aerogel-fibre-based thermal renders were investigated and also compared with the benchmark solutions taking into account a broader climatic range.

## 2. Inputs for Numerical Simulation

### 2.1. Simulated Facade Multilayer Walls

**Figure 1:**
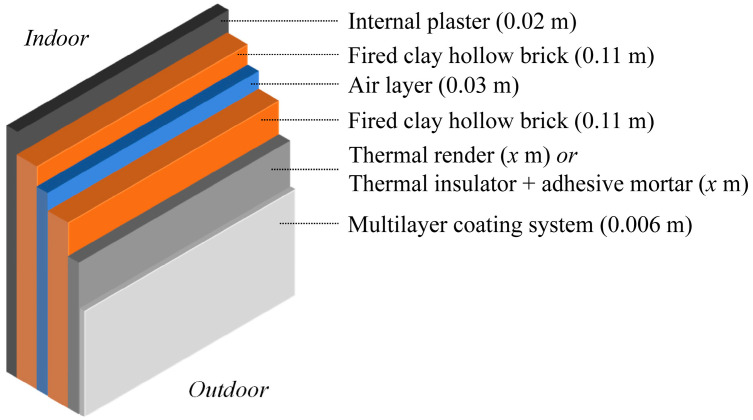
Double-leaf wall composition for facade retrofitting.

A double-leaf composition with an air cavity was chosen to simulate the facade walls, following the retrofit strategies for Portuguese buildings from the 1960s to the 1980s [27], which represent around 2,000,000 buildings in need of retrofitting [28]. Figure 1 depicts the simulated double-leaf assembly specifying its component layers.

As shown in Figure 1, the aerogel-fibre-based thermal renders are directly applied on the surface of the fired clay hollow brick substrate [29], with a maximum thickness of 0.08 m, as prescribed by the manufacturers. Other thermal insulation materials, such as EPS and XPS, need an extra layer of adhesive mortar (≈0.005 m) to bind them to the substrate, which was included in the simulations.

A finishing multilayer coating system was applied over the thermal renders or the benchmark thermal insulation materials. This multilayer coating comprised commercially available products: a base coat, a fibreglass mesh, a key coat, and an acrylic finishing coat, resulting in a total thickness of ≈0.006 m; this finishing system was previously characterised in terms of environmental impacts [30]. The finishing layer, the internal plaster, and the fired clay hollow bricks were the same in all the simulated cases, and their contribution was only considered in the solutions’ thermal transmittance (U). Table 1 shows the main properties of the layers on the double-leaf wall used in all the simulated cases.

### 2.2. Aerogel-Fibre-Based Thermal Renders

The aerogel-based thermal renders studied by Pedroso et al. [21,32] with aramid (TR aramid) and sisal fibres (TR sisal) with, respectively, 0.50% and 0.10% substitution quantities concerning the reference render powder (in total volume) were used in the numerical simulations [33]. Furthermore, thermal renders without fibres were considered the reference (TR reference). The choice of aramid and sisal fibres for the numerical simulations was based on previous studies reporting their contribution to fundamental properties related to aerogel-based thermal renders, such as mechanical strength, especially when compared with reference renders; aramid fibres performed better than polypropylene regarding mechanical and thermal performance, and sisal fibres led to more relevant differences in the fresh state, mechanical and physical properties, impact resistance, economics, and LCA compared to forest biomass fibres [20,32,33]. Sisal vegetal fibres benefit from a lower industrialisation need [34]. The fibres’ characterisation can be checked in [32].

Two scenarios were investigated regarding the costs of the formulations. The non-optimised scenario refers to the cost of the thermal renders considering production prices gathered with an aerogel thermal render manufacturer, and the optimised scenario relates to the consideration of the decrease in the silica aerogel market cost that is expected shortly, as evaluated in Pedroso et al. [33]. Table 2 shows the non-optimised and optimised costs for each studied thermal render.

For life cycle assessments (LCAs), results on the abiotic depletion potential from fossil fuels (ADP−ff) and global warming potential (GWP) were based on the authors’ previous work [20]. Furthermore, similar processes available in the Ecoinvent 3 database (v3.4) [35], present in the SimaPro software (v8.5.2.0), were adopted and compared to Jelle [30]. Table 3 presents properties of the aerogel-fibre-based thermal renders used in the numerical simulations.

### 2.3. Thermal Insulation Benchmark Solutions

The benchmark solutions evaluated against aerogel-fibre-based thermal renders are the most commonly used in retrofitting with available data [30,36,37]: EPS, XPS, MW, and ICB, as well as TR cork. Although other thermal renders may present lower declared thermal conductivities, their environmental indicators are not yet available. The selected solutions enabled similar wall compositions, and, as mentioned before, in the case of non-render thermal insulators, an additional adhesive mortar layer was required. Table 4 shows some main properties of the benchmark solutions used in the numerical simulations.

Table 5 presents the Ecoinvent 3 processes and literature references for the simulations concerning environmental characterisation and a reference for the adhesive mortar.

### 2.4. Climate

Following Pedroso et al. [33], the Azores (altitude: 48 m; longitude: 25°40′ W; latitude 37°43′ N) and Bragança (altitude: 674 m; longitude: 6°45′ W; latitude 41°48′ N) regions in Portugal were selected for simulation and assessment of the aerogel-fibre-based thermal renders, due to their substantially varied climatic conditions. The Azores should lead to fewer energy demands by showing a more temperate characterisation; on the other hand, Bragança has the coldest climate, with an intermediate need for cooling, resulting in higher energy demands. The Azores have a Cfb Köppen climate classification, referring to a temperate maritime climate, and Bragança has a Csb class, with a temperate climate with dry summer and mild temperatures [42,43,44].

Several studies adopted the concept of degree-days (DD) [45,46,47] to optimise thermal insulation thicknesses for buildings and, consequently, achieve energy conservation. DD may be divided into cooling (CDD) and heating degree-days (HDD). CDD and HDD enable the assessment of the expected relative differences in heating and cooling energy requirements for a specific building submitted to different climates [48] regarding indoor reference temperatures for the cooling and heating seasons. In Portugal, reference indoor temperatures are 25 °C and 18 °C, respectively, for the cooling and heating seasons [49], with a cooling season of 4 months [50]. Thus, for the Azores, HDD and CDD are, respectively, 604 °C·day and 0 °C·day and for Bragança, 2015 °C·day and 136 °C·day [49,50,51]. As the present study refers to retrofitting scenarios, only the heating degree-days (HDD) were accounted for since older buildings usually achieve cooling through natural ventilation [52].

A range of HDD values was adopted for general assessments of the aerogel-fibre-based thermal render performance and the benchmark analysis, providing a broadened discussion, including different regions in Portugal and, possibly, worldwide.

### 2.5. Calculation Parameters

Table 6 presents information concerning the electricity mix used in Portugal and considered in the numerical simulations.

Indoor and outdoor air film thermal resistance (R_in_ and R_out_) inputs for the simulations were 0.13 and 0.04, respectively [54]. Interest and inflation rates were 3.0% and 2.0%, without considering risks [28]. The energy efficiency of the heating (COP) was considered as 1.00, referring to the frequent use of electric radiators [55] in retrofitted buildings. The service life of the simulated facades was considered to be 30 years. Maintenance actions were disregarded since all the solutions had the same finishing coating, leading to similar expected impacts on the outer layer.

The benchmark study included a first approach regarding optimised thicknesses of the cost-optimised aerogel-fibre-based thermal renders compared to all benchmark materials as a function of varied HDD values, aiming to encompass several regions in Portugal. The second assessment approach considered a fixed 0.02 m thickness for the insulation layer, pursuing the achievement of thermal transmittance (U) legal requirements. The recurrent thickness of renders is 0.02 m in facades of old buildings [27] and aims to minimise influences on their architectural aspect. Concerning legal requirements, for small retrofit interventions, with cost below 25% of the building’s value, the expected thermal transmittance values depend on the buildings’ geographical location, being U ≤ 1.70 W∙m^−2^∙°C^−1^ for I1 regions in Portugal, U ≤ 1.50 W∙m^−2^∙°C^−1^ for I2 regions, and U ≤ 1.40 W∙m^−2^∙°C^−1^ for I3 regions. In the remaining cases, legal requirements are similar to the new construction ones [56,57]. For the simulated scenarios, the original uninsulated wall was the reference.

## 3. Numerical Simulation Modelling

Numerical simulation modelling followed the methodology used by Pedroso et al. [33]. Firstly, annual energy consumption for heating (E_cons,heat_) was determined [33,46,58] regarding HDD values, thermal transmittances of the studied wall systems (U), energy efficiency ratio COP, and, lastly, the lower heating value of the energy source (H_u_), following Equation (1).

(1) $$E_{cons,heat}=\frac{86400\cdot U\cdot HDD}{COP\cdot H_{u}}$$

where H_u_ was considered 3.60 × 10^6^ J∙kW∙h^−1^ for electricity.

Secondly, within the economic analysis, savings during the studied wall systems’ service life (S_SL_) [33,46,59,60,61,62,63] were determined with Equation (2).

(2) $$S_{SL}=\left(\frac{86400\cdot \left(U_{un}-U_{ins}\right)\cdot HDD}{COP\cdot H_{u}}\right)\cdot C_{e}\cdot PWF-C_{ins}$$

where U_un_ and U_in_ correspond to the thermal transmittance [W∙m^−2^∙°C^−1^] of the thermally uninsulated and insulated walls, respectively, C_e_ is the energy cost for electricity [€∙kW^−1^∙h^−1^], PWF is the present worth factor, and, lastly, C_ins_ is the cost associated with the thermal insulation material, per unit area [€∙m^−2^].

Still, regarding economic assessments, thermal insulation optimum thicknesses (x_opt_) were calculated with Equation (3) [58,60,64].

(3) $$x_{opt}=293.94\cdot {\left(\frac{HDD\cdot C_{e}\cdot PWF\cdot \lambda_{ins}}{COP\cdot H_{u}\cdot C_{i}}\right)}^{\frac{1}{2}}-R_{wt}\cdot \lambda_{ins}$$

where λ_ins_ corresponds to the thermal conductivity of the materials [W·m^−1^·K^−1^], C_i_ to the cost of the thermal insulation materials per cubic meter [€∙m^−3^], and R_wt_ [m^2^·K·W^−1^] to the sum of R_in_, R_w_ (the wall thermal resistance without thermal insulation), and R_out_.

Furthermore, the calculation of payback periods (PP) for the thermal insulation materials, within the economic analysis, followed Equation (4) [60,61].

(4) $$PP=\frac{C_{ins}}{S_{ES}}$$

where S_ES_ corresponds to the annual energy cost savings [€∙m^−2^∙year^−1^] obtained by the difference between the annual costs of the thermally insulated and uninsulated walls [46,47,65].

Finally, the environmental evaluation of the thermal insulating materials regarded their thickness multiplied by the environmental impacts for each cubic meter, considering ADP−ff and GWP. Then, the environmental payback of the solutions (PP_ADP_−_ff OR GWP_) [year] for the 30-year service life was determined with Equation (5) [46,66].

(5) $${PP}_{ADP-ffORGWP}=\frac{I_{ins}}{I_{solwithoutins}-I_{solwithins}}$$

where I_ins_ is the environmental impact of the thermal insulation material for a given thickness, I_sol without ins_ corresponds to the impacts of the energy consumption in an uninsulated wall, and I_sol with ins_ to the impacts of the energy consumption in an insulated wall. ADP−ff and GWP units are [MJ∙m^−2^∙year^−1^] and [kg CO_2_ eq∙m^−2^∙year^−1^], respectively. The optimised environmental impact thickness corresponds to the lowest sum of impacts throughout the service life.

## 4. Results and Discussion

### 4.1. Aerogel-Fibre-Based Thermal Renders

Table 7 presents the main results of the numerical simulations obtained for the studied aerogel-fibre-based thermal renders regarding non-optimised and optimised (opt) cost scenarios.

As expected, since Bragança has much higher heating needs (HDD), the optimum thermal render thicknesses are also higher, contributing to much higher economic, energy, and environmental savings when compared with the Azores (≈230 €∙m^−2^, ≈4000 MJ∙m^−2^, and ≈400 kg CO_2_ eq∙m^−2^). Cuce et al. [65] also reported increasing aerogel optimum insulation thicknesses for rising degree-days.

The U-value was not a primary concern for this specific comparison regarding legal requirements. So, in the Azores, the requirements of U ≤ 0.45 W∙m^−2^∙°C^−1^ [67] were not fulfilled; on the other hand, in Bragança, TR_opt_ aramid and TR_opt_ sisal accomplished the legal demands (U ≤ 0.35 W∙m^−2^∙°C^−1^ [67]). Aerogel-based renderings may be used to ensure thermal regulation requirements relating to energy efficiency, especially in poorly insulated old houses, reducing heating loads by up to 50%; generally, in new buildings, heating reductions are lower if their exterior envelope is already insulated [68].

**Figure 2:**
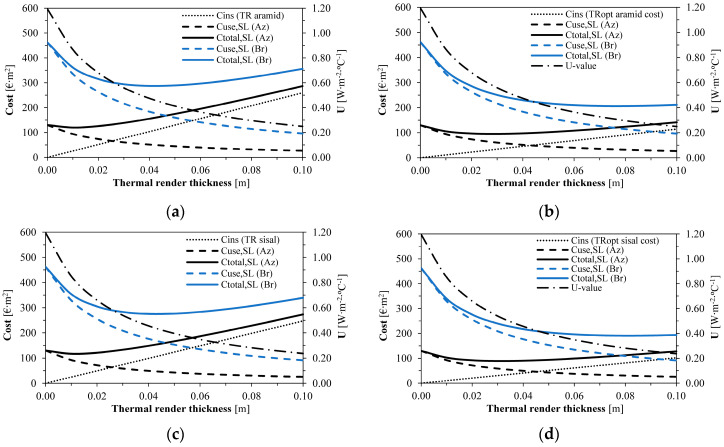
Optimum insulation thickness versus cost for: (**a**) TR aramid; (**b**) TR_opt_ aramid; (**c**) TR sisal; (**d**) TR_opt_ sisal. Caption: Az: Azores and Br: Bragança.

As shown in Table 7 and Figure 2, the cost-optimised formulations significantly increased the thermal render optimum thicknesses (from 0.01 to 0.03 m in the Azores and from 0.04 to 0.08 m in Bragança). Due to lower energy needs in the Azores since they present more stable temperatures throughout the year, a higher impact resulted from the thermal render costs during the service life. The TR_opt_ sisal formulation led to the best economic performance among all the aerogel-fibre-based enhanced formulations.

**Figure 3:**
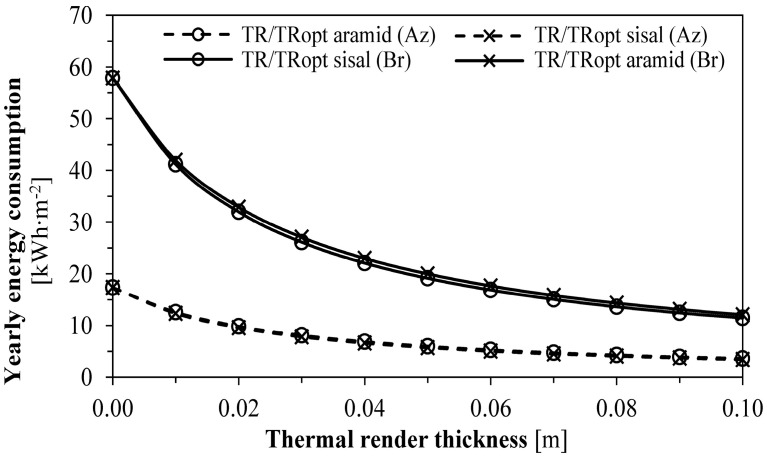
Annual energy consumption. Caption: Az: Azores and Br: Bragança.

Figure 3 depicts the annual energy consumption of the optimised and non-optimised aerogel-fibre-based thermal renders. For the most demanding climate, in Bragança, the thermal insulation thickness presented a higher influence on the energy consumption reduction than in the least demanding climate. For instance, 0.01 m of thermal render can lead to an annual saving of 16.67 kWh∙m^−2^ in Bragança, while only 4.80 kWh∙m^−2^ in the Azores.

**Figure 4:**
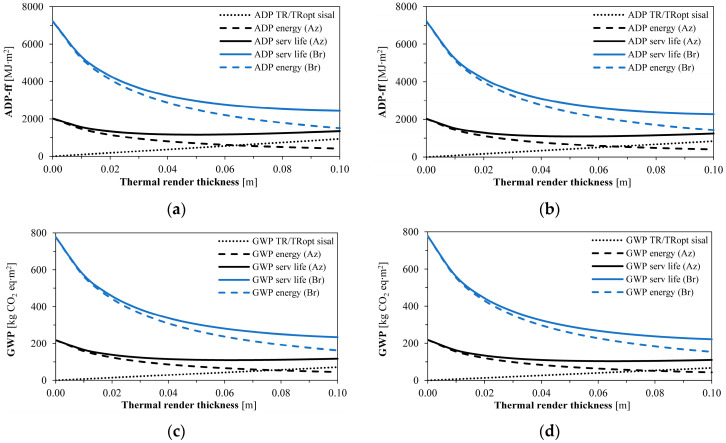
Environmental indicators for the different thicknesses of the aerogel-fibre-based thermal renders: (**a**) ADP−ff for TR aramid and TR_opt_ aramid; (**b**) ADP−ff for TR sisal and TR_opt_ sisal; (**c**) GWP for TR aramid and TR_opt_ aramid; (**d**) GWP for TR sisal and TR_opt_ sisal. Caption: Az: Azores and Br: Bragança.

The environmental indicators ADP−ff and GWP (Table 7 and Figure 4) indicate that the higher the HDD, the higher the impact that energy had on the results. With the increase in the thermal insulation thickness, the energy impacts decrease since less energy is necessary to keep the same indoor climatic conditions.

The environmental impacts of the thermal renders linearly increased with their thickness. Since the Azores present the lowest HDD, the energy impacts were also lower than for Bragança, with less influence on the overall environmental performance. As depicted in Figure 4, the optimum thickness to pursue the lowest ADP−ff and GWP impacts throughout the service life is increased compared to the economic optimum thickness, resulting in 0.05 m for the Azores and more than the manufacturers’ technical limit of 0.08 m in Bragança.

**Figure 5:**
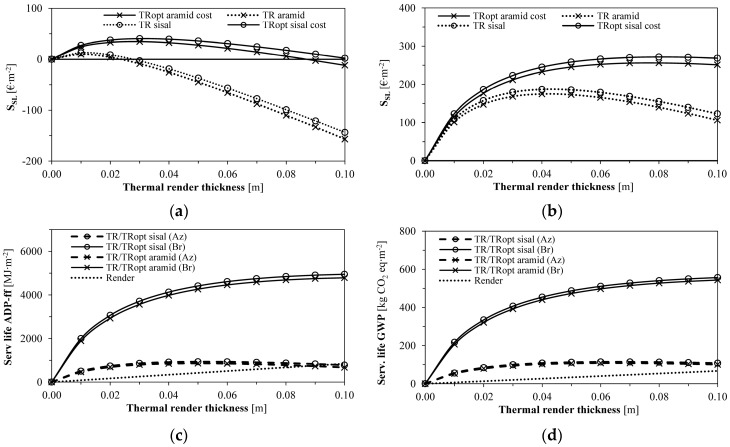
Service life impact savings: (**a**) Economic: Azores; (**b**) Economic: Bragança; (**c**) Environmental: ADP−ff; (**d**) Environmental: GWP. Caption: Az: Azores and Br: Bragança.

Figure 5 corroborates that the highest economic service life savings (S_SL_) are found in Bragança for different thermal render thicknesses due to the higher HDD values. Nonetheless, in the Azores, it is also possible to obtain savings (as the ≈40 €∙m^−2^ vs. ≈270 €∙m^−2^ in Bragança for x_opt_). In terms of minimising the environmental impacts, increased thermal insulation thicknesses lower the heating energy consumption; furthermore, the incorporated impacts are possibly more diluted than energy impacts compared to the economic assessment.

Pedroso et al. [20] identified that 95% of the environmental impacts related to silica-aerogel-fibre-based thermal renders are due to the raw materials, among which the synthesis of silica aerogel represents more than 90% because of the use of isopropanol and electricity for drying. Therefore, there is still an imperative need to decrease the negative environmental impacts associated with silica aerogel synthesis; for example, by recycling reagents involved in the production and reducing the drying time, silica aerogel environmental impacts could be reduced by more than 85% [20], supporting its use in thermal renders. Furthermore, Garrido et al. [69] reported that cost reductions at the industrial scale may be achieved by replacing supercritical drying with drying under atmospheric pressure. Moreover, regarding toxicity, although silica-based aerogel has biomedical applications with reports on its biocompatibility [70,71], Vareda et al. [72] referred to the high number of nanoparticles that may be released by silica aerogel handling, leading to dry skin and upper respiratory tract irritation and, thus, emphasising the need for personal protective equipment and ventilation.

**Figure 6:**
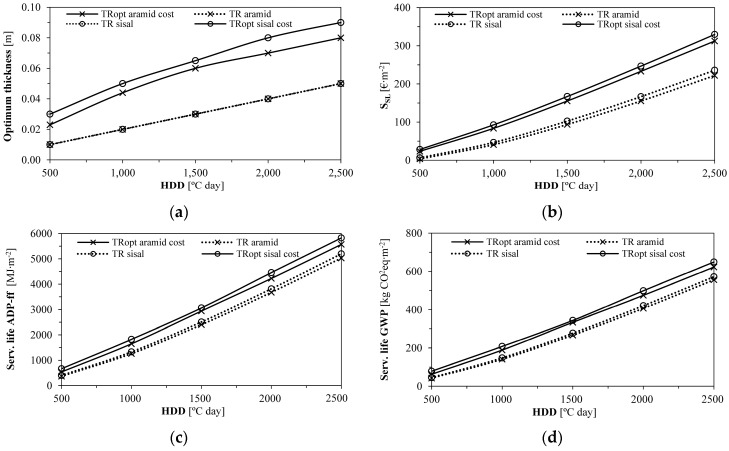
Influence of the HDD on the optimum thermal render thickness and the respective economic and environmental impacts: (**a**) Optimum thickness as a function of HDD; (**b**) Service life economic savings (S_SL_) for the optimum thickness; (**c**) Service life ADP−ff savings for the optimum thickness; (**d**) Service life GWP savings for the optimum thickness.

Figure 6 enables the evaluation of different HDD scenarios for comparison with other regions than the Azores and Bragança. As expected, an increase in the HDD led to higher optimum thicknesses, decreasing the U-values of the walls. Then, economic and environmental savings may be obtained for the optimum thickness related to each HDD in all locations, being higher in the more demanding ones. Again, TR_opt_ sisal showed the best overall performance.

Figure 6 suitably covers the Portuguese territory by referring to HDD between 500 °C·day and 2500 °C·day. However, additional simulations would be required to expand the assessment and investigate the aerogel-fibre-based thermal render applicability exposed to more extreme climates, such as those from Sweden, Finland, and Norway, which present the HDD energy indicator with values near 5000 °C·day [73]. Moreover, additional research should consider the recommendation by Cuce et al. [65] for using the investigated insulation material in colder climates regarding investments with longer lifetimes. The aerogel-based thermal renders which perform best regarding life cycle economic and energy costs are not always the best in terms of the initial investment [24].

### 4.2. Benchmark Analysis

Table 8 presents the individual results for the numerical simulations within the benchmark analysis regarding the optimum thicknesses found for the aerogel-fibre-based thermal renders as a function of different HDD values.

**Figure 7:**
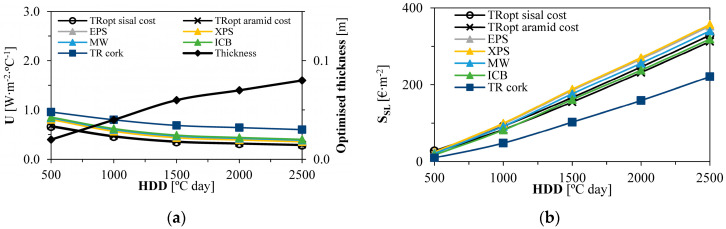

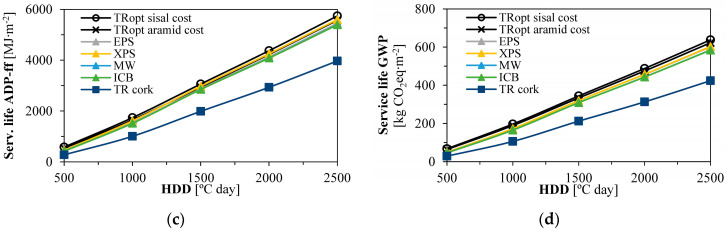
Influence of the HDD on the optimum thermal insulation thicknesses and the respective economic and environmental impacts: (**a**) U-value and optimum thickness as a function of HDD; (**b**) Service life economic savings (S_SL_) for each thermal insulation material optimum thickness; (**c**) Service life ADP−ff savings for each thermal insulation material optimum thickness; (**d**) Service life GWP savings for each thermal insulation material optimum thickness.

Then, Figure 7a shows that, with increasing HDD and rising heating energy demand, thermal insulation optimum thickness increases. Lower energy costs can mitigate this increment in thickness and the resulting insulation costs. Figure 7a further depicts that the lowest U-value is associated with TR_opt_ sisal, which improves the building users’ comfort compared to the other solutions; on the other hand, the highest U-value refers to TR cork: this behaviour is due to the low thermal conductivity presented by the aerogel-fibre-based thermal renders.

Figure 7b shows that the cost-optimised aerogel-fibre-based thermal renders provide similar savings to ICB and MW insulation solutions (≈320 €∙m^−2^ at 2500 HDD), with TR cork showing the lowest savings during the considered service life (≈220 €∙m^−2^ at 2500 HDD). Regarding the environmental impacts, in Figure 7c,d, TR cork showed the lowest savings again, while, with increasing HDD, TR_opt_ aramid and TR_opt_ sisal started to slowly show higher savings than some of the other benchmark materials, for instance, 5740 (TR_opt_ sisal) vs. 5600 (XPS), or 5390 (ICB) MJ∙m^−2^ and approximately 635 (TR_opt_ sisal) vs. 600 (EPS) or 580 (ICB) kg CO_2_ eq∙m^−2^, both for 2500 HDD.

Although XPS was the material with the highest economic savings (8% more savings than TR_opt_ sisal, at 2500 HDD), TR_opt_ sisal showed 3% higher savings of ADP−ff and 6% of GWP compared to XPS (for 2500 HDD), resulting in a better environmental performance.

Table 9 presents the individual results for the second simulated approach within the benchmark assessment, with thermal insulation thickness fixed at 0.02 m.

**Figure 8:**
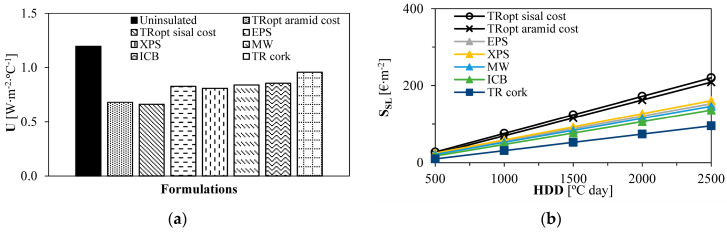

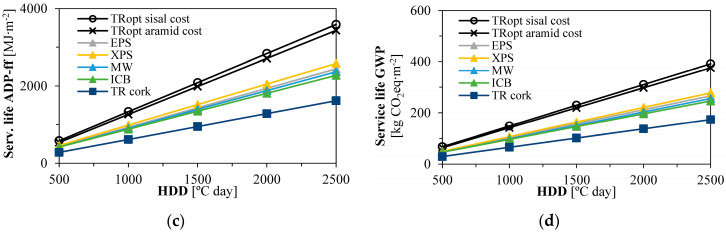
Performance comparison considering the same thermal insulation thickness (0.02 m) and different HDD: (**a**) U-values for each thermal insulation material; (**b**) Service life economic savings (S_SL_) for each thermal insulation material; (**c**) Service life ADP−ff savings for each thermal insulation material; (**d**) Service life GWP savings for each thermal insulation material.

Figure 8 depicts that for the same thermal insulation thickness, the aerogel-fibre-based cost-optimised renders still show the lowest U-value compared to other benchmark solutions. With 0.02 m, it is possible to lower the U-value from 1.20 W∙m^−2^∙°C^−1^ on the original uninsulated wall to 0.66 W∙m^−2^∙°C^−1^, significantly improving building users’ comfort and dropping energy needs (≈50% improvement). This is suitable considering that Garrido et al. [24] identified that economic savings could not be achieved for walls retrofitted with aerogel thermal mortars if the compensations were of only 10% in heating and cooling needs compared to reference walls, mainly due to transportation and efforts on the renders’ application. All the other insulation products presented higher U-values and, therefore, contributed less to the comfort of the indoor environment; XPS, for instance, led to a U-value of ≈0.80 W∙m^−2^∙°C^−1^.

In this context, Cuce et al. [65], studying aerogel-based thermal superinsulation, although not focused on renders, stated that their use resulted in lower thicknesses than conventional materials. Ibrahim et al. [74] also reported that the difference in the thickness of aerogel plasters compared to other insulation plasters was between 7 cm and 20 cm to retrofit an exterior envelope from a U-value of 6.4 W/m^2^·K^−1^ to 0.4 W/m^2^·K^−1^; compared with polystyrene and glass wool, the difference was lower, within 2 cm to 3.5 cm.

For a small thickness of thermal insulation, Figure 8 shows that TR_opt_ aramid and TR_opt_ sisal showed the highest economic and environmental savings from 1000 HDD onwards, an attractive solution for facade retrofitting. The aerogel-fibre-based renders represent, for 2500 HDD, savings of around EUR 60∙m^−2^, 1000 MJ∙m^−2^, and 100 kg CO_2_ eq∙m^−2^, for a 30-year service life, compared to the second-best performing material (XPS). Higher HDD will possibly lead to even higher savings. Again, the TR cork showed the lowest economic and environmental savings.

Therefore, the results demonstrate the potential of using silica-aerogel-fibre-based thermal renders compared to benchmark solutions in several situations. However, the potential of aerogel innovative materials may be further regarded as energy-saving when additionally incorporated into other elements of building envelopes, for instance, glass units for windows [75,76] or glass bricks [77]. In the case of windows, they may be responsible for 30% to 50% of heat loss and gain in buildings [78], thus affecting energy consumption. So, an integrated application of silica aerogel incorporated in thermal insulation and other facade elements could expand the resulting benefits.

## 5. Conclusions

This paper discussed the application of silica-aerogel-based thermal renders with aramid and sisal fibres for retrofitting in facade walls, compared to usual benchmark solutions. In the context of global warming and climate change, thermal retrofitting of the building stock may contribute to its environmental and energy performance, also supporting economic sustainability.

Aerogel-fibre-based thermal renders were assessed with the Azores and Bragança climate conditions. Higher heating needs (HDD) shown by Bragança resulted in higher optimum thermal render thicknesses and enhanced economic, energy, and environmental savings. In the Azores, the thermal render costs during the considered service life substantially impacted the results. Given the expected near-future behaviour of the aerogel market, its cost optimisation increased the thermal renders’ optimum thicknesses. TR_opt_ sisal formulations led to the best performance. Furthermore, the annual energy consumption reduced more when increasing thermal render thicknesses in the most demanding climate: 0.01 m of thermal render may provide an annual saving of 16.67 kWh∙m^−2^ in Bragança and 4.80 kWh∙m^−2^ in the Azores. Regarding environmental impacts, the higher the HDD, the higher the impact of energy on the ADP−ff and GWP indicators.

Considering different regions, aerogel thermal renders with aramid and sisal fibres and benchmark solutions presented higher thermal insulation optimum thicknesses with increasing HDD. Within the first studied approach in benchmark analysis, TR cork, EPS, XPS, MW, and ICB were evaluated. The lowest thermal transmittance (U) was associated with TR_opt_ sisal, and TR cork had the highest U-value. Cost-optimised aerogel-fibre-based thermal renders provided economic savings similar to ICB and MW (≈320 €∙m^−2^ at 2500 HDD), while XPS had the best results. On the other hand, TR_opt_ sisal had a better environmental performance with increasing HDD; for 2500 °C·day, 5740 vs. 5600 (XPS) or 5390 (ICB) MJ∙m^−2^, and approximately 635 vs. 600 (EPS) or 580 (ICB) kg CO_2_ eq∙m^−2^. Therefore, although XPS was the most economical (8% more savings than TR_opt_ sisal, at 2500 HDD), TR_opt_ sisal had 3% more ADP−ff and 6% GWP savings.

In the second retrofitting approach, with a fixed 0.02 m thickness for thermal insulation, the U-value was lowered from 1.20 W∙m^−2^∙°C^−1^ for the original uninsulated wall to 0.66 W∙m^−2^∙°C^−1^ with TR_opt_ sisal. Aerogel-fibre-based thermal renders significantly outperformed the benchmark materials for HDD of 1000 °C∙day onwards. As such, they can save approximately EUR 60∙m^−2^, 1000 MJ∙m^−2^, and 100 kg CO_2_ eq∙m^−2^ throughout the 30-year lifetime when compared with the second-best material: XPS. TR cork led to the lowest economic and environmental savings because of its higher thermal conductivity. Aerogel renders’ thermal insulation counterbalanced their embodied impacts (per m^3^) regarding the decrease in energy consumption.

Further research is suggested using silica aerogel in thermal insulation and other facade elements, such as windows and glass units. In addition, substantiated by the present paper, simulations of more extreme climates, with higher HDDs than 2500 °C·day, should be carried out.

## Figures and Tables

**Table 1:** Properties of the layers on the simulated double-leaf wall.

Material	Thickness [m]	R [m^2^∙K∙W^−1^]
Air layer [31]	0.030	0.150
Fired clay hollow brick [31]	0.110	0.250
Internal plaster [31]	0.020	0.011
Multilayer coating system [29,30]	0.0055	0.007

**Table 2:** Costs of the aerogel-fibre-based thermal renders regarding non-optimised and optimised scenarios [33].

Formulation	Non-Optimised Scenario	Optimised Scenario
TR reference	2478 €∙m^−3^	1021 €∙m^−3^
TR aramid	2596 €∙m^−3^	1146 €∙m^−3^
TR sisal	2477 €∙m^−3^	1021 €∙m^−3^

**Table 3:** Properties of the aerogel-fibre-based thermal renders [33].

Material	Density [kg∙m^−3^]	λ [W∙m^−1^∙K^−1^]	ADP−ff [MJ∙m^−3^]	GWP [kg CO_2_ eq∙m^−3^]
TR aramid	165	0.032	9303.2	720.4
TR sisal	160	0.030	8452.3	672.2

**Table 4:** Properties of the benchmark thermal insulation solutions.

Material	Density [kg∙m^−3^]	Thickness [m]	λ [W∙m^−1^∙K^−1^]	R [m^2^∙K∙W^−1^]	Cost [€∙m^−3^]	ADP−ff [MJ∙m^−3^]	GWP [kg CO_2_ eq∙m^−3^]
Adhesive mortar [30]	1300	0.005	0.450	0.011	1040.0 *	4797.0	455.0
EPS [11,31]	20		0.040		65.8 *	2800.0	111.0
ICB [31,38]	110		0.045		333.6 *	821.0	40.2
MW [11,31]	150		0.042		158.0 *	2295.0	208.5
TR cork [39]	825		0.095		566.0 *	2739.0	333.3
XPS [31,40]	30		0.037		118.0 *	2625.0	291.9

Note: * costs obtained by averaging the prices of several distributors.

**Table 5:** Processes and literature references used for the environmental characterisation of the benchmark thermal insulation materials.

Material	Ecoinvent Processes and Literature References
EPS	EPS insulation board at plant/kg [11]
ICB	[38,40]
Mineral wool	Stone wool, packed {GLO} | market for | Cut-off, S [11]
XPS	Polystyrene, extruded {GLO} | market for | Cut-off, S [11]
TR cork	[41]
Adhesive mortar	[30]

**Table 6:** Characteristics of the electricity mix in Portugal [33,35,53].

Ecoinvent Process	Cost [€∙kWh^−1^]	ADP−ff [MJ∙kWh^−1^]	GWP [kg CO_2_ eq∙kWh^−1^]	Lower Heating Value (H_u_) [J∙kW∙h^−1^]
Electricity, low voltage {PT} | market for | Cut-off, S	0.22	3.90	0.42	3.60 × 10^6^

**Table 7:** Optimum insulation thicknesses, economic and environmental impacts, service life (SL) savings (S_SL_), and payback periods (PP) for the aerogel-fibre-based thermal renders. Results for non-optimised and optimised (opt) cost scenarios.

Region	Thermal Render	U [W∙m^−2^∙°C^−1^]	x_opt_ [m]	C_ins_ [€∙m^−2^]	S_SL_ [€∙m^−2^]	PP [year]	SL ADP−ff Savings [MJ∙m^−2^]	PP ADP−ff [year]	SL GWP Savings [kg CO_2_ eq∙m^−2^]	PP GWP [year]
Azores	TR aramid	0.86	0.01	25.96	9.74	>30	464.80	5.00	52.87	3.60
	TR sisal	0.85	0.01	24.77	12.65	>30	500.22	4.34	56.25	3.20
	TR_opt_ aramid	0.56	0.03	34.40	34.71	29.7	800.59	7.75	94.66	5.58
	TR_opt_ sisal	0.53	0.03	30.65	40.57	22.6	859.17	6.84	99.67	5.05
Bragança	TR aramid	0.47	0.04	103.84	174.86	17.8	3982.13	2.56	440.12	1.84
	TR sisal	0.45	0.04	99.09	186.82	15.9	4128.83	2.27	454.16	1.68
	TR_opt_ aramid	0.30	0.08	91.72	256.06	10.7	4689.30	4.11	527.52	2.95
	TR_opt_ sisal	0.28	0.08	81.74	271.61	9.0	4844.26	3.67	540.73	2.71

**Table 8:** Optimum insulation thicknesses as a function of the HDD, costs, and environmental impact savings.

Material Designation	HDD [°C·day]	U [W∙m^−2^∙°C^−1^]	x_opt_ [m]	C_ins_ [€∙m^−2^]	S_SL_ [€∙m^−2^]	PP [year]	SL ADP−ff Savings [MJ∙m^−2^]	PP ADP−ff [year]	SL GWP Savings [kg CO_2_ eq∙m^−2^]	PP GWP [year]
TR_opt_ aramid	500	0.68	0.02	22.93	23.43	29.4	538.3	10.4	63.6	6.8
	1000	0.47	0.04	45.86	83.71	16.4	1652.0	6.8	189.2	4.6
	1500	0.36	0.06	68.79	155.22	13.3	2941.7	5.7	333.7	3.9
	2000	0.33	0.07	80.26	232.05	10.4	4228.1	4.6	475.1	3.2
	2500	0.30	0.08	91.72	312.49	8.8	5570.9	4.0	622.5	2.8
TR_opt_ sisal	500	0.66	0.02	20.43	27.67	22.2	582.5	8.7	67.5	6.0
	1000	0.46	0.04	40.87	92.05	13.3	1738.6	5.8	196.8	4.1
	1500	0.35	0.06	61.30	167.14	11.0	3062.0	5.0	344.1	3.5
	2000	0.31	0.07	71.52	246.31	8.7	4374.0	4.1	488.0	2.9
	2500	0.28	0.08	81.74	328.94	7.5	5740.0	3.5	638.0	2.5
TR cork	500	0.96	0.02	11.40	9.84	34.7	279.0	5.9	29.0	6.9
	1000	0.80	0.04	22.80	48.15	14.2	1006.0	3.3	106.0	3.8
	1500	0.69	0.06	34.20	102.83	10.0	1988.0	2.5	212.0	2.8
	2000	0.64	0.07	39.90	159.16	7.5	2934.0	2.0	313.0	2.2
	2500	0.60	0.08	45.60	221.13	6.2	3969.0	1.7	424.0	1.9
EPS	500	0.83	0.02	8.19	24.04	10.2	427.7	5.3	49.4	3.0
	1000	0.59	0.04	9.51	97.69	2.9	1541.8	2.6	173.0	1.2
	1500	0.46	0.06	10.83	185.45	1.8	2876.0	2.0	320.0	0.9
	2000	0.41	0.07	11.49	266.75	1.3	4128.0	1.6	457.0	0.7
	2500	0.37	0.08	12.15	352.55	1.0	5450.0	1.4	601.0	0.6
XPS	500	0.81	0.02	9.00	24.97	10.8	457.0	4.8	49.0	4.6
	1000	0.57	0.04	11.40	99.85	3.4	1612.0	2.3	173.0	2.3
	1500	0.44	0.06	13.80	188.19	2.2	2976.0	1.8	320.0	1.8
	2000	0.39	0.07	15.00	270.50	1.7	4255.0	1.4	458.0	1.5
	2500	0.35	0.08	16.20	357.12	1.4	5600.0	1.2	603.0	1.3
MW	500	0.84	0.02	9.60	21.57	13.4	419.0	4.8	46.0	4.1
	1000	0.60	0.04	12.80	91.86	4.2	1521.0	2.2	165.6	1.9
	1500	0.47	0.06	16.00	176.66	2.7	2850.0	1.7	309.5	1.4
	2000	0.42	0.07	17.60	256.01	2.1	4092.0	1.3	443.5	1.1
	2500	0.39	0.08	19.20	339.97	1.7	5405.0	1.1	585.0	1.0
ICB	500	0.85	0.02	12.23	17.48	21.0	418.0	3.3	46.0	2.5
	1000	0.62	0.04	18.93	82.15	6.9	1517.0	1.2	165.0	0.8
	1500	0.49	0.06	25.63	161.83	4.8	2850.0	0.8	309.0	0.5
	2000	0.44	0.07	28.98	237.96	3.7	4083.0	0.6	443.0	0.4
	2500	0.40	0.08	32.33	318.85	3.0	5391.0	0.5	584.0	0.3

**Table 9:** Thermal insulation thickness fixed at 0.02 m as a function of the HDD: costs and environmental impact savings.

Material Designation	HDD [°C·day]	U [W∙m^−2^∙°C^−1^]	x [m]	C_ins_ [€∙m^−2^]	S_SL_ [€∙m^−2^]	PP [year]	SL ADP−ff Savings [MJ∙m^−2^]	PP ADP−ff [year]	SL GWP Savings [kg CO_2_ eq∙m^−2^]	PP GWP [year]
TR_opt_ aramid	500	0.68	0.02	22.93	23.43	29.4	538.3	10.4	63.6	6.8
	1000				69.80	9.9	1262.7	4.4	141.6	3.1
	1500				116.16	5.9	1987.0	2.8	219.6	2.0
	2000				162.53	4.2	2711.4	2.1	297.6	1.5
	2500				208.89	3.3	3435.8	1.6	375.6	1.2
TR_opt_ sisal	500	0.66		20.43	27.67	22.2	582.5	8.7	67.5	6.0
	1000				75.77	8.1	1334.0	3.8	148.4	2.7
	1500				123.87	4.9	2085.5	2.4	229.4	1.8
	2000				171.97	3.6	2837.0	1.8	310.3	1.3
	2500				220.07	2.8	3588.5	1.4	391.2	1.0
TR cork	500	0.96		11.40	10.02	34.1	279.7	5.9	29.3	6.9
	1000				31.44	10.9	614.4	2.7	65.4	3.1
	1500				52.86	6.5	949.0	1.7	101.4	2.0
	2000				74.29	4.6	1283.7	1.3	137.5	1.5
	2500				95.71	3.6	1618.4	1.0	173.5	1.2
EPS	500	0.83		8.19	24.04	10.2	427.6	5.3	49.4	3.0
	1000				56.28	4.4	931.2	2.4	103.6	1.4
	1500				88.51	2.8	1434.8	1.6	157.8	0.9
	2000				120.74	2.0	1938.4	1.2	212.1	0.7
	2500				152.98	1.6	2442.0	0.9	266.3	0.5
XPS	500	0.81		9.00	24.97	10.8	457.7	4.8	49.6	4.6
	1000				58.93	4.6	988.4	2.2	106.7	2.1
	1500				92.90	2.9	1519.1	1.4	163.9	1.4
	2000				126.87	2.1	2049.8	1.1	221.0	1.0
	2500				160.84	1.7	2580.5	0.8	278.2	0.8
MW	500	0.84		9.60	21.57	13.4	419.3	4.8	46.1	4.1
	1000				52.74	5.5	906.3	2.2	98.6	1.9
	1500				83.92	3.4	1393.4	1.5	151.0	1.3
	2000				115.09	2.5	1880.4	1.1	203.5	0.9
	2500				146.26	2.0	2367.4	0.9	255.9	0.7
ICB	500	0.85		12.22	17.48	21.0	418.5	3.3	46.2	2.5
	1000				47.19	7.8	882.6	1.5	96.2	1.2
	1500				76.89	4.8	1346.8	1.0	146.2	0.8
	2000				106.60	3.4	1810.9	0.8	196.1	0.6
	2500				136.31	2.7	2275.0	0.6	246.1	0.5

## Data Availability

Not applicable.
